# Risk factors for failure to return to the pre-fracture place of residence after hip fracture: a prospective longitudinal study of 444 patients

**DOI:** 10.1007/s00402-012-1469-8

**Published:** 2012-02-07

**Authors:** Anne J. H. Vochteloo, Sabine T. van Vliet-Koppert, Andrea B. Maier, Wim E. Tuinebreijer, Maarten L. Röling, Mark R. de Vries, Rolf M. Bloem, Rob G. H. H. Nelissen, Peter Pilot

**Affiliations:** 1 Department of Orthopaedic Surgery, Leiden University Medical Centre, P.O. Box 9600, 2300 RC Leiden, The Netherlands; 2 Department of Orthopaedic Surgery, Reinier de Graaf Group, Delft, The Netherlands; 3 Department of Gerontology and Geriatrics, Leiden University Medical Centre, Leiden, The Netherlands; 4 Department of Surgery-Traumatology, Erasmus MC, University Medical Centre, Rotterdam, The Netherlands; 5 Department of Surgery, Reinier de Graaf Group, Delft, The Netherlands

**Keywords:** Hip fracture, Place of residence, Risk factors, Longitudinal

## Abstract

**Introduction:**

Long-term place of residence after hip fracture is not often described in literature. The goal of this study was to identify risk factors, known at admission, for failure to return to the pre-fracture place of residence of hip fracture patients in the first year after a hip fracture.

**Methods:**

This is a prospective longitudinal study of 444 consecutive admissions of hip fracture patients aged ≥65 years. Place of residence prior to admission, at discharge, after 3 and 12 months was registered. Patients admitted from a nursing home (*n* = 49) were excluded from statistical analysis. Multivariable logistic regression analysis was performed, using age, gender, presence of a partner, ASA-score, dementia, anaemia at admission, type of fracture, pre-fracture level of mobility and level of activities of daily living (ADL) as possible risk factors.

**Results:**

Two hundred eighty-nine patients lived in their own home, 31.8% returned at discharge, 72.9% at 3 months and 72.8% at 12 months. Age, absence of a partner, dementia, and a lower pre-fracture level of ADL or mobility were independent contributors to failure to return to their own home at discharge, 3 or 12 months. 106 patients lived in a residential home; 33.3% returned at discharge, 68.4% at 3 months and 64.4% at 12 months. Age was an independent contributor to failure to return to a residential home.

**Conclusions:**

Age, dementia and a lower pre-fracture level of ADL were the main significant risk factors for failure to return to the pre-fracture residence. As the 3- and 12-month return-rates were similar, 3-month follow-up might be used as an endpoint in future research.

## Introduction

The total number of hip fracture patients aged 50 years and older has been estimated to increase to over half a million in the US by 2040 and 6.3 million by 2050 worldwide [1, 2]. Elderly hip fracture patients suffer frequently from comorbidities and the 1-year mortality rate is high [3]. Social morbidity as measured by limited activities of daily life, loss of independency and the impact of a sudden change in place of residence due to a hip fracture has little focus in research, despite its importance for the quality of life for the patient [4]. Socio-economically, the impact of a hip fracture and its sequelae is large as well. Discharge to an alternative location or arranging additional postoperative care at home after discharge can attribute to a longer stay in hospital and creates additional costs [5, 6]. Only a limited number of prospective studies on pre- and post-fracture place of residence have been published. Most of them are focused on the discharge location, but not on the long-term place of residence [7–12].

The aim of the current study was to identify risk factors, known at admission, for failure to return to the pre-fracture place of residence at discharge, 3- and 12-month post-fracture. These factors can be used to improve discharge protocols and could become important socio-economic parameters.

## Methods

### Patient cohort

A prospective longitudinal observational cohort study of 444 consecutive admissions for a hip fracture in 437 patients of 65 years and older was conducted. All patients were admitted to the orthopaedic or trauma surgery ward in a 450-bed teaching hospital in Delft, the Netherlands, from January 2008 to December 2009. In both wards professionals worked with a standardized care pathway for hip fracture patients that has been developed by a multidisciplinary team, including orthopaedic and trauma surgeons, geriatricians, psychiatrists and nurses from the emergency department, wards and liaison service. Patients with a fracture due to a high-energy trauma, with a pathologic fracture or those with a periprosthetic hip fracture were not included. Patients admitted from a nursing home (*n* = 49, 12-month mortality 46.9%) were excluded from the analysis for failure to return to their pre-fracture nursing home as they all returned to the nursing home or died. Length of follow-up for all patients was 12 months or up to death.

It was not necessary to obtain approval from the local ethical committee due to the observational character of the study evaluating usual care as a part of good clinical practice. Since data could not be traced back to the individual patient, there were no privacy issues.

### Data collection

Uniform collection and recording of data of all patients were achieved by standard evaluation at admission and after 3 and 12 months according to the standardized care pathway for hip fracture patients. Age, gender, presence of a partner, American Society of Anesthesiologists (ASA) physical status classification score, presence of dementia, presence of anaemia at admission, type of fracture, fracture treatment, anaesthesia, length of stay (LOS), discharge location and the in-hospital, 3- and 12-month mortality rate were registered [13]. One observer (AV) rated the ASA score of all patients. Mortality of the patients was scored meticulously by repeated consultation of the population registers of the counties in the region as well as the hospital’s patient registration systems for the full length of follow-up.

Place of residence, level of mobility and level of activities of daily living (ADL) were obtained at admission and at 3- and 12-month post-fracture [14]. These parameters were registered during routine follow-up in the outdoor clinic or by a questionnaire sent to the patient or caretakers in case of dementia.

### Anaemia

In all patients the haemoglobin level at admission was obtained. Anaemia at admission was defined based on the criteria of the World Health Organization (WHO) [15]. These criteria classify anaemia as a haemoglobin level below 7.5 mmol/L (12 g/dL) in women and below 8.1 mmol/L (13 g/dL) in men.

### Place of residence

Patients were divided into three groups based on pre-fracture place of residence at admission, i.e. living in their own home, in a residential home or in a nursing home.

Living in their own home was defined as living independently, alone or with a partner.

A residential home is a heterogeneous form of living, ranging from the availability of support to almost full-time help in daily activities.

A nursing home is a residential facility caring for persons with predominant difficulties in activities of daily living.

### Level of mobility and ADL

Mobility both in- and outdoors prior to hip fracture was classified as mobile without an aid, mobile with an aid or not able to ambulate (“immobile”). A cane, crutch(es) or walker were all considered an aid, patients in a wheelchair were considered to be immobile. The level of mobility was divided into four main categories; mobile without use of an aid in- and outdoors, mobile in- and outdoors with the use of an aid in- and/or outdoors, only mobile indoors (regardless the use of an aid) and immobile both in- and outdoors.

The Groningen Activity Restriction Score (GARS) is a functional ADL score [14]. It assesses competence in abilities in 11 personal basic ADL and 7 instrumental activities of daily living (IADL). A summed score was calculated ranging from 18 (indicating ability to perform all activities without assistance or undue effort) to 72 (indicating disability).

### Statistical analysis

Continuous data are presented as mean with standard deviations (SD). The independent Student’s *t* test or one-way anova was used to compare groups of continuous data. Categorical data are presented as the number of subjects in the category, along with the percentages. Chi-square test and Fisher’s exact test were used for comparing groups of categorical data.

Bivariate and multivariable logistic regression analysis was performed to identify risk factors for patients living in their own home or in a residential home prior to admission for failure to return to the pre-fracture place of residence at discharge, 3- and 12-month post-fracture. For both analysis, only risk factors known at admission were used; age, gender, presence of a partner, perioperative risk (ASA score I/II vs. III/IV), dementia, anaemia at admission, pre-fracture level of mobility (using the four categories of mobility), pre-fracture level of ADL (expressed with the GARS) and type of fracture [neck of femur (inter) trochanteric or subtrochanteric].

Patients classified as ASA I or II and III or IV were combined to two groups, as the separate groups of ASA I (*n* = 22) and ASA IV (*n* = 26) classified patients were too small to be analyzed separately. LOS was changed into a binary summary outcome based on the median, i.e. ≤ or >11 days.

The likelihood ratio backward test was used to find the best-fit model by selecting the variables one by one. The probability for entry was set at 0.05, and the probability for removal at 0.10. To calculate odds ratios (OR), logistic regression analysis was used. *P* values lesser than 0.05 were considered statistically significant. All data were analysed in SPSS 17.0 (SPSS Inc. Chicago, USA).

## Results

Table 1 shows characteristics of all patients, based on the pre-fracture place of residence. Mean (SD) age of all patients was 83.4 years (7.3), 73.2% were female. Prior to hip fracture, the majority of patients (*n* = 289, 65.1%) lived in their own home and nearly one quarter (*n* = 106, 23.9%) lived in a residential home. A small group (*n* = 49, 11.0%) resided in a nursing home. Patients living in their own home were younger, more often male, had lower ASA scores, were less often known with anaemia and dementia and had more often a partner compared to patients living in a residential home or a nursing home.

**Table 1:** Characteristics of patients with a hip fracture dependent on residency at admission

	Study population (*n* = 444)	Place of residence at admission			*P* value*
		Own home (*n* = 289)	Residential home (*n* = 106)	Nursing home (*n* = 49)	
Mean age in years (SD)	83.4 (7.3)	81.9 (6.9)	86.9 (7.4)	85.2 (6.2)	<0.001
Female gender	325 (73.2)	200 (69.2)	87 (82.1)	38 (77.6)	0.010
Partner at admission^a^	133 (32.4)	116 (40.4)	11 (10.8)	6 (27.3)	<0.001
ASA score					
I/II	289 (65.1)	205 (70.9)	59 (55.7)	25 (51.0)	<0.001
III/IV	155 (34.9)	84 (29.1)	47 (44.3)	24 (49.0)	
Dementia^b^	111 (25.9)	31 (11.2)	44 (42.3)	36 (76.6)	<0.001
Anaemia at admission	188 (42.5)	105 (36.5)	52 (49.5)	31 (63.3)	<0.001
Mobility at admission					<0.001
Without an aid in- and outdoors	152 (34.3)	133 (46.0)	14 (13.2)	5 (10.4)	
With an aid in- and outdoors	200 (45.1)	136 (47.1)	46 (43.4)	18 (37.5)	
Only mobile indoors	78 (17.6)	18 (6.2)	42 (39.6)	18 (37.5)	
Immobile	13 (2.9)	2 (0.7)	4 (3.8)	7 (14.6)	
Mean GARS (SD)^c^	42.9 (17.8)	34.8 (14.5)	55.3 (13.5)	63.7 (9.0)	<0.001
Fracture type					
Neck of femur	257 (57.9)	169 (58.5)	57 (53.8)	31 (63.3)	0.140
(Inter) trochanteric	169 (38.1)	106 (36.7)	45 (42.5)	18 (36.7)	
Subtrochanteric	18 (4.1)	14 (4.8)	4 (3.8)	0 (0)	
Treatment					
Osteosynthesis	248 (55.9)	162 (56.1)	64 (60.4)	22 (44.9)	0.093
(Hemi) arthroplasty	184 (41.4)	120 (41.5)	38 (35.8)	26 (53.1)	
Conservative	12 (2.7)	7 (2.4)	4 (3.8)	1 (2.0)	
Anaesthesia					
Spinal/epidural	406 (91.4)	268 (92.7)	94 (88.7)	44 (89.8)	0.302
General	26 (5.9)	14 (4.8)	8 (7.5)	4 (8.2)	
Not applicable	12 (2.7)	7 (2.4)	4 (3.8)	1 (2.0)	
LOS >10 days	209 (47.1)	133 (46.0)	62 (58.5)	14 (28.6)	<0.001
Mortality					
In-hospital	20 (4.5)	6 (2.1)	10 (9.4)	4 (8.2)	0.004
3 months	67 (15.1)	22 (7.6)	28 (26.4)	17 (34.7)	<0.001
3- to 12-month	50 (11.3)	26 (9.0)	18 (17.0)	6 (12.2)	0.017
12 months	117 (26.4)	48 (16.6)	46 (43.4)	23 (46.9)	<0.001

Values are given as number (percentage) unless mentioned otherwise

*LOS* length of stay, *GARS* Groningen Activity Restriction Score, *ASA* American Society of Anesthesiologists Physical Status classification

Data not available in:^ a^ 33 patients,^ b^ 15 patients,^ c^ 7 patients

*Bivariate analysis

A conservative treatment was chosen in 12 patients, who therefore did not receive any form of anaesthesia.

Mortality at 3 months was 15.1% (*n* = 67) and 26.4% (*n* = 117) at 12 months of the entire group, being higher in the group of patients that lived less independently. At 3-month follow-up, no data about place of residence were available in 13 patients (2.8%), and at 12-month follow-up this information was missing in 6 patients (1.4%).

Data on some variables were missing (most of them less than 4%, Table 1), since they were not entered in the prospective database at admission and could not be retrieved at a later period in time.

### Patients living in their own home at admission

Discharge directly to their own home occurred in 90 patients (31.8%). At 3 months, 186 patients (72.9%) and at 12 months 171 patients (72.8%) returned to their own home. All values were corrected for mortality; in-hospital mortality was 2.1% (*n* = 6), mortality at 3 months was 7.6% (*n* = 22) and 16.6% (*n* = 48) at 12 months.

#### Risk factors for failure to return to the own home

**Fig. 1:**
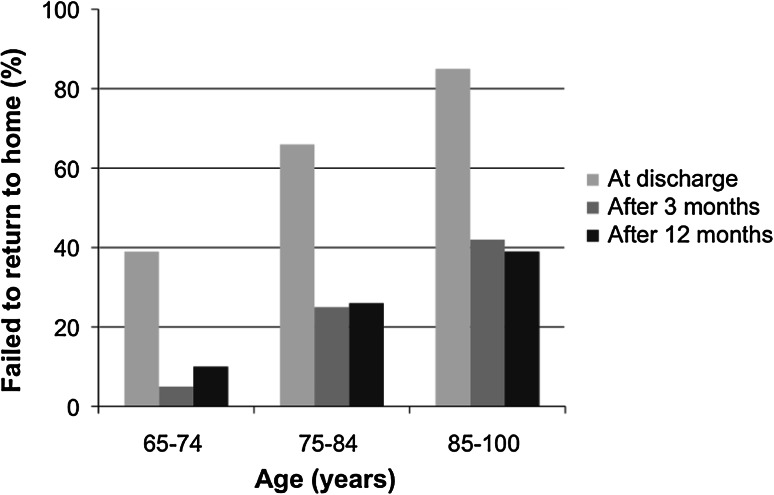
Percentages of patients who failed to return to their own home at discharge, 3  and 12 months

Data of the bivariate regression analysis for risk factors for failure to return to home are shown in Table 2. Age, absence of a partner, dementia, a lower level of mobility and a lower level of ADL (i.e. a higher GARS) were significant contributors to failure to return to their own home at discharge, at 3 and at 12 months after discharge. Figure 1 shows the positive association of chronological age and failure of returning home at discharge and after 3 and 12 months. Multivariable logistic regression analysis showed that age, dementia and a lower level of ADL (i.e. a higher GARS) were the main significant independent contributors to failure to return to their own home at discharge, at 3 or at 12 months, as demonstrated in Table 3. Absence of a partner was a significant risk factor for failure to return to their own home only at discharge.

**Table 2:** Characteristics of patients living in their own home at admission

	Discharge to own home		*P* ^#^	At 3 months back at own home		*P* ^#^	At 12 months back at own home		*P* ^#^
	Yes (*n* = 90)	No (*n* = 193)		Yes (*n* = 186)	No (*n* = 69)		Yes (*n* = 171)	No (*n* = 64)	
Mean age in years (SD)	77.9 (6.8)	83.6 (6.2)	*	80.0 (6.5)	85.1 (6.0)	*	79.9 (6.5)	84.6 (5.8)	*
Female gender	47 (52.2)	148 (76.7)	*	127 (68.3)	47 (68.1)		115 (67.3)	47 (73.4)	
Partner at admission	53 (59.6)	59 (30.7)	*	85 (45.7)	20 (29.0)	*	83 (48.5)	18 (28.1)	*
ASA score III/IV	21 (23.3)	58 (30.1)		44 (23.7)	22 (31.9)		32 (18.7)	23 (35.9)	*
Dementia	2 (2.3)	29 (15.6)	*	7 (3.9)	19 (29.2)	*	8 (4.8)	15 (25.4)	*
Anaemia at admission	27 (30.0)	73 (38.0)		60 (32.4)	24 (34.8)		48 (28.2)	23 (35.9)	
Pre-fracture mobility			*			*			*
Without an aid in- and outdoors	61 (67.8)	70 (36.3)		102 (54.8)	19 (27.5)		101 (59.1)	15 (23.4)	
With an aid in- and/or outdoors	28 (31.1)	105 (54.4)		77 (41.4)	42 (60.9)		65 (38.0)	42 (65.6)	
Cannot walk outside	1 (1.1)	16 (8.3)		6 (3.2)	7 (10.1)		3 (1.8)	7 (10.9)	
Immobile	0 (0)	2 (1.0)		1 (0.5)	1 (1.4)		2 (1.2)	0 (0.0)	
Mean GARS (SD)	27.2 (12.3)	38.0 (14.3)	*	38.1 (13.0)	60.7 (10.6)	*	35.3 (13.5)	58.8 (11.0)	*
Fracture type			*						
Neck of femur	62 (68.9)	105 (54.4)		116 (62.4)	38 (55.1)		107 (62.6)	36 (56.3)	
(Inter) trochanteric	23 (25.6)	79 (40.9)		62 (33.3)	26 (37.7)		56 (32.7)	24 (37.5)	
Subtrochanteric	5 (5.6)	9 (4.7)		8 (4.3)	5 (7.2)		8 (4.7)	4 (6.3)	

Values are given as number (percentage), * Significant at that moment in time,^ #^ *P* < 0.05, bivariate analysis

*ASA* American Society of Anesthesiologists Physical Status classification, *GARS* Groningen Activity Restriction Score

**Table 3:** Risk factors known at admission for failing to return to their own home

	Independent variables	Odds ratio	95% CI	*P* value
At discharge^a^	Age (per year)	1.10	1.05 to 1.16	<0.001
	Female gender	2.23	1.17 to 4.26	0.015
	Absence of a partner	2.00	1.06 to 3.78	0.032
	Dementia	4.84	1.02 to 23.0	0.047
	GARS (per 10 units)	1.48	1.16 to 1.89	0.002
At 3 months^b^	Age (per year)	1.10	1.03 to 1.16	0.003
	Dementia	9.21	3.14 to 27.0	<0.001
	GARS (per 10 units)	1.84	1.42 to 2.35	<0.001
At 12 months^c^	Age (per year)	1.09	1.03 to 1.16	0.003
	Dementia	5.96	2.23 to 15.9	<0.001
	Mobility category^d^			
	With an aid in- and/or outdoors	2.97	1.39 to 6.32	0.005
	Only mobile indoors	6.03	1.39 to 26.2	0.016

Multivariable logistic regression analysis

Analysis performed in^ a^ 268 patients,^ b^ 242 patients;^ c^ 225 patients

^d^Reference category is mobile without an aid

*GARS* Groningen Activity Restriction Score

### Patients living in a residential home at admission

Discharge directly to their residential home occurred in 32 patients (33.3%). At 3 months 54 (68.4%) and at 12 months 38 (64.4%) patients were residing in their pre-fracture residential home again. All values were corrected for mortality; in-hospital mortality was 9.4%, mortality was 26.4% at 3 months and 43.4% at 12 months.

#### Risk factors for failure to return to the residential home

Data of the bivariate analysis for risk factors for failure to return to a residential home are shown in Table 4. A lower level of ADL (higher GARS) was a risk factor for failure to return to the residential home at 3- and at 12-month post-fracture. Age and female gender were risk factors at discharge.

**Table 4:** Characteristics of patients living in a residential home at admission

	Discharge to residential home		*P* ^#^	At 3 months back at residential home		*P* ^#^	At 12 months back at residential home		*P* ^#^
	Yes (*n* = 32)	No (*n* = 64)		Yes (*n* = 54)	No (*n* = 25)		Yes (*n* = 38)	No (*n* = 21)	
Mean age in years (SD)	83.5 (9.0)	88.1 (6.2)	*	85.2 (8.3)	86.9 (5.6)		86.0 (7.7)	86.8 (6.7)	
Female gender	22 (68.8)	57 (89.1)	*	44 (81.5)	22 (88.0)		34 (89.5)	18 (85.7)	
Partner at admission	1 (3.2)	9 (14.5)		8 (15.1)	1 (4.2)		7 (18.9)	2 (10.0)	
ASA score III/IV	12 (37.5)	29 (45.3)		17 (31.5)	12 (48.0)		11 (28.9)	9 (42.9)	
Dementia	17 (53.1)	23 (37.1)		21 (40.4)	11 (44.0)		16 (44.4)	8 (38.1)	
Anaemia at admission	12 (38.7)	34 (53.1)		24 (45.3)	10 (40.0)		19 (50.0)	10 (47.6)	
Pre-fracture mobility									
Without an aid in- and outdoors	5 (15.6)	9 (14.1)		9 (16.7)	2 (8.0)		7 (18.4)	3 (14.3)	
With an aid in- and/or outdoors	14 (43.8)	29 (45.3)		25 (46.3)	10 (40.0)		19 (50.0)	7 (33.3)	
Only mobile indoors	12 (37.5)	26 (40.6)		20 (37.0)	12 (48.0)		12 (31.6)	10 (47.6)	
Immobile	1 (3.1)	0 (0.0)		0 (0.0)	1 (4.0)		0 (0.0)	1 (4.8)	
Mean GARS (SD)	55.2 (14.6)	54.0 (13.0)		59.7 (11.8)	66.3 (8.0)	*	58.1 (12.8)	66.8 (8.1)	*
Fracture type									
Neck of femur	17 (53.1)	34 (53.1)		27 (50.0)	15 (60.0)		21 (55.3)	10 (47.6)	
(Inter) trochanteric	14 (43.8)	29 (45.3)		26 (48.1)	10 (40.0)		17 (44.7)	10 (47.6)	
Subtrochanteric	1 (3.1)	1 (1.6)		1 (1.9)	0 (0.0)		0 (0.0)	1 (4.8)	

Values are given as number (percentage), * Significant at that moment in time,^ #^ *P* < 0.05, bivariate analysis

*ASA* American Society of Anesthesiologists Physical Status classification, *GARS* Groningen Activity Restriction Score

Multivariable logistic regression analysis (96 patients available) showed that age was the only independent contributor to failure to return to their residential home at discharge (OR 1.09, 95% CI 1.02–1.16, *P* = 0.007). None of the other potential risk factors reached significance at discharge, at 3- or at 12-month follow-up. The latter two analyses were performed in 79 and 59 patients, respectively.

## Discussion

The majority of the hip fracture patients in the studied population aged 65 years and older lived in their own home, whilst sustaining a hip fracture. During the first year after fracture treatment, three quarters of the surviving population had returned to their own home. Multivariable logistic regression analysis identified higher age, dementia and a lower level of mobility as the most important risk factors for failure to return to their own home at discharge, but also at 3- and at 12-month post-fracture.

The percentage of patients returning to their pre-fracture residence was stable between 3  and 12 months, the 3-month time mark can be used as an evaluation end point in future research. This is in line with a previous study which concluded that the 4-month time mark is adequate to evaluate ADL and residential status in hip fracture patients [16].

The medical and social morbidity of patients living in a residential home was worse compared to patients living in their own home, this is reflected in a higher 1-year mortality rate and a more limited level of mobility of the residential home patients. The overall 1-year mortality rate in our cohort (mean age 83 years) was 26%, comparable to the result of an US study (495 patients, mean age 85 years, 1-year mortality 26%) and a large Scottish cohort (27,475 patients, aged 50 and older, 1-year mortality 31%) [17, 18].

Early and reliable information on the potential discharge location after the hospital admission is of importance for both patients and caregivers to plan postoperative care. Furthermore, it is of socio-economical impact. When extrapolating the results of this study to different countries, one must be aware of bias at several levels. First, large differences between countries exist in type of housing and traditions for homes for elderly people [19]. In the Netherlands, a residential home is very heterogeneous form of living, as defined earlier in this paper. Secondly, large regional, national and international differences exist on discharge policies, like locations of discharge and availability of different kinds of temporary rehabilitation units [20, 21].

In a large Scottish series, the number of hip fracture patients living in their own home prior to sustaining a hip fracture and the percentage of these patients returning to this location after 4 months were comparable to our results [22]. In a series of hip fracture patients living in New York slightly more (85%) patients lived in their own residence prior to the hip fracture, but only 20% could be immediately discharged home from the hospital. At 6 months, 83% had returned back to their own residence [10]. Finally, in a comparison of a Finnish and a British cohort of hip fracture patients, 62 and 69% lived in their own home prior to the hip fracture, of whom 44 and 54% respectively, had returned home four  months after hospital discharge, which is less than that in our study [20].

Risk factors for not returning to their own home after hospital discharge were higher age, presence of dementia, absence of a partner, a lower level of mobility and a lower level of ADL prior to the hip fracture. Beside these risk factors, a longer LOS was also a risk factor for failure to return to their own home. A longer LOS is often associated with a higher rate of adverse outcomes during admission and might be related to worse outcome thereafter [23, 24]. LOS was not included in the statistical analysis, since the purpose of the current study was to identify risk factors known at admission of the patient. Subsequently, a prediction model for discharge location already at admission can be developed with these risk factors. If discharged to another location other than the patient’s own home, length of hospital stay is usually longer, with subsequent additional costs [5, 6]. An instrument that predicts the discharge location already at admission would therefore be of great value, not only for liaison services but also for patients and their family.

Advanced age, dementia, a walking disability and concomitant chronic systemic diseases were previously reported to be risk factors for failure to return to the patient’s own home at discharge from hospital [7]. This is largely in concordance with our findings, although we used a more general categorisation for scoring the overall degree of comorbidities (ASA classification). But, others have shown that ASA score and the number and type of comorbidities are associated [13]. We found a higher ASA score to be a significant risk factor in the bivariate analysis, but not in the multivariable logistic regression analysis. Other papers identified presence of a partner, good general health, good cognition, a higher level of ADL and mobility (both pre-fracture and 2 weeks after surgery), a lower number of medication, and moderate use of nursing interventions (like bathing) as important variables predicting discharge to an own residence [7–10, 25, 26]. These findings are in line with our study. Other risk factors like anaemia at admission and fracture type were of small importance in the bivariate analysis and lost significance in the multivariable analysis. This is most probably because both anaemia and fracture type are strongly correlated to age, as shown in previous papers [27–32].

Deakin et al. [9] published the largest series (3,240 patients) on discharge location of hip fracture patients. Their analyses were not specified for pre-fracture place of residence. Pre-injury dependence, age, male gender and injury sustained whilst in hospital were identified as the main risk factors for discharge to an alternative location (DAL). In contrast to their findings, we identified female gender as an independent risk factor for DAL This conflicting outcome is most probably due to difference in sample size between our and the former study. Furthermore, we only included patients admitted for a hip fracture at the emergency department; no patients with an in-hospital hip fracture were included.

Some limitations exist; first, the analyses of patients living in a residential home at admission were troubled by the limited numbers and a high mortality rate. At the 3-month follow-up, 86 patients could be analyzed; at 12 months only 66 patients. This is the main reason we could only identify age as a risk factor for failure to return to their residential home at discharge in the multivariate analysis. A lower level of ADL independency was the most important risk factor for not returning to their residential home in the bivariate analysis. A second limitation was the fact that the diagnosis of dementia was based on medical history. Cognitive performance was not assessed during hospital stay. The third limitation was that the type and number of comorbidities were not registered. Finally, many factors influence the location of residence after hospital discharge. Of these other factors, the role of the social network might be one of the largest. They can play an important role in the decision of an older person whether or not to stay living independently or to move to a residential home.

In conclusion, the large study population, the prospective character, adequate information on mortality rates and long follow-up make the study results valuable for analysis of socio-economic aspects after hip fracture treatment, especially for the patients living in their own home prior to hip fracture. This study identified higher age, dementia and a lower pre-fracture level of ADL as the most important independent risk factors for failure to return to the pre-fracture residence in patients living in their own home prior to hip fracture. In residential home patients, age was identified as the only risk factor, possibly due to the small patient numbers.

We will use these risk factors to develop a model that predicts discharge location at admission to provide better information for patients, family and physicians regarding the discharge and rehabilitation process.
